# Transcriptome Analysis Revealed the Symbiosis Niche of 3D Scaffolds to Accelerate Bone Defect Healing

**DOI:** 10.1002/advs.202105194

**Published:** 2022-01-18

**Authors:** Ce Ji, Minglong Qiu, Huitong Ruan, Cuidi Li, Liang Cheng, Juan Wang, Changwei Li, Jin Qi, Wenguo Cui, Lianfu Deng

**Affiliations:** ^1^ Department of Orthopaedics Shanghai Key Laboratory for Prevention and Treatment of Bone and Joint Diseases Shanghai Institute of Traumatology and Orthopaedics Ruijin Hospital Shanghai Jiao Tong University School of Medicine 197 Ruijin 2nd Road Shanghai 200025 P. R. China

**Keywords:** 3D printed scaffolds, endochondral ossification, molecular network, symbiotic microenvironment, tissue repair

## Abstract

Three dimension (3D) printed scaffolds have been shown to be superior in promoting tissue repair, but the cell‐level specific regulatory network activated by 3D printing scaffolds with different material components to form a symbiosis niche have not been systematically revealed. Here, three typical 3D printed scaffolds, including natural polymer hydrogel (gelatin‐methacryloyl, GelMA), synthetic polymer material (polycaprolactone, PCL), and bioceramic (*β*‐tricalcium phosphate, *β*‐TCP), are fabricated to explore the regulating effect of the symbiotic microenvironment during bone healing. Enrichment analysis show that hydrogel promotes tissue regeneration and reconstruction by improving blood vessel generation by enhancing oxygen transport and red blood cell development. The PCL scaffold regulates cell proliferation and differentiation by promoting cellular senescence, cell cycle and deoxyribonucleic acid (DNA) replication pathways, accelerating the process of endochondral ossification, and the formation of callus. The *β*‐TCP scaffold can specifically enhance the expression of osteoclast differentiation and extracellular space pathway genes to promote the differentiation of osteoclasts and promote the process of bone remodeling. In these processes, specific biomaterial properties can be used to guide cell behavior and regulate molecular network in the symbiotic microenvironment to reduce the barriers of regeneration and repair.

## Graphical abstract

Three typical 3D printed scaffolds, including hydrogel, synthetic polymer, and bioceramic, are fabricated to explore the regulating effect of the symbiotic microenvironment during bone healing via transcriptome analysis. Specific biomaterial properties can be used to guide cell behavior and regulate molecular network in the symbiotic microenvironment to reduce the barriers of regeneration and repair.

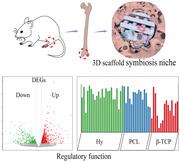

## 1 Introduction

In recent years, ribonucleic acid sequencing (RNA‐Seq) was widely used in biology and medicine filed. As an effective detection method, RNA‐Seq played an important role in deciphering the structure and function of genomes, identifying genetic networks in cells, physiological, biochemical, and biological systems, and establishing molecular biomarkers for diseases, pathogens, and environmental challenges.^[^ ^1^ ^]^ The global cognition of gene expression, signal transduction regulatory networks, cell states, and biological functions in biological processes were of great significance for the identification of maker genes and transcription factors in development,^[^ ^2^ ^]^ regeneration,^[^ ^3^ ^]^ and disease process.^[^ ^4^ ^]^ With the development of regenerative medicine and biomedical engineering, biomaterials have been developed to accelerate tissue regeneration and repair.^[^ ^5^ ^]^ In addition to its role as support and mechanical fixation, the ability to guide cell behavior has been a central concept to fabricate biomaterials. Using biomaterials to deliver therapeutic drugs, cytokines, small molecules, micro‐RNAs, growth factors, or stem cells in damaged tissues, the regenerated processes were improved by regulating cell migration, proliferation, differentiation of mesenchymal stem cells, polarization of macrophage, and production of Treg cells.^[^ ^6^ ^]^ The varied biomaterials were applied in the fields of angiogenesis, neurogenesis, wound healing, bone regeneration, and antimicrobial therapies.^[^ ^7^ ^]^ At present, the interaction of biomaterials and surrounding tissue after implantation still needed to further reveal (such as different components of biological materials could make active different degrees of cell adhesion, proliferation, differentiation, inflammatory response,^[^ ^8^ ^]^ and tissue microenvironment). It restricts the selection of suitable biomaterials for tissue repair from the perspective of the symbiotic microenvironment composed of material and organism.

The development of 3D printing technology makes it possible for the spatial geometric configuration and internal structure design of biomaterials. According to the injury tissue structure, biomaterials can be fabricated into a specific 3D scaffold and applied to large bone defects, fracture healing, oral repair, and skin injury repair.^[^ ^9^ ^]^ Especially in the field of bone injury repair, based on bone shape and injury shape, 3D scaffold materials can be designed, fabricated and applied under different conditions.^[^ ^10^ ^]^ Although kinds of 3D scaffold materials are applied in the repair process of bone injury by providing mechanical support or bionic structure,^[^ ^11^ ^]^ and have shown a good characteristic of bone healing, the specific mechanism and the change of cell function activated by biomaterials are different. For example, the hydrogel scaffolds can promote bone regeneration through early recruitment of mesenchymal stem cells.^[^ ^12^ ^]^ The bionic bone scaffolds with complete Haversian bone structure improve the residence capacity of cells through structural changes, thus achieving the function of promoting blood vessel growth and new bone shape in vivo.^[^ ^13^ ^]^ Layered porous hydroxyapatite/tricalcium phosphate scaffolds promote bone regeneration by promoting the adhesion and proliferation of osteoblasts.^[^ ^14^ ^]^ The Polycaprolactone (PCL) scaffolds with bionic meniscus structure are applied in cartilage regeneration,^[^ ^15^ ^]^ and composite 3D hydrogel‐ceramic scaffolds enhance bone and cartilage regeneration by improving the binding interface.^[^ ^16^ ^]^ However, the specificity raised cell proliferation and differentiation and the regulation mechanism triggered by different components biomaterials are still not clear,^[^ ^17^ ^]^ which also limits the further application of 3D scaffolds materials.^[^ ^18^ ^]^ The effect of different biomaterials on the cell behavior and cell function of the same injured tissue will be the main basis for selecting suitable biomaterials for tissue injury repair. As a symbiosis niche composed by 3D scaffold, cells, and regenerated tissues, it accelerates the bone healing process. Understanding the changes of genes expression, signal transduction, regulating network, and cell behavior in the symbiosis niche during the regeneration process, is meaningful to further fabricate and apply of 3D scaffold for bone healing.

The understanding and regulation of signal transduction network to match the bone healing stages will help to improve the efficiency of injury repair and function recovery. The repair of bone injury is a postnatal regenerative process that involves various cell types and signal transduction during the whole regeneration processes.^[^ ^19^ ^]^ It mainly underwent three major stages of inflammatory response, endochondral ossification, and bone remodeling.^[^ ^20^ ^]^ In the inflammatory stage, M1 macrophages, neutrophils, leukocytes, mesenchymal stem cells, and M2 macrophages are mainly involved. In endochondral ossification stage, fibrocytes, chondrocytes, osteoblasts, and hematopoietic stem cells are mainly involved. Osteoblasts, osteoclasts, and osteocytes are mainly involved in bone remodeling stage.^[^ ^21^ ^]^ Transcriptional level regulation determines cell state and the initial of main phases. Under transcriptome regulation, mesenchymal stem cells (MSCs) which *Sox9* gene is activated and highly express will differentiate into chondrocytes, but the MSCs highly express *Runx2* will differentiate into osteoblasts. The chondrocytes which the expression of *Pth1r*, *Ihh*, *Col10a1*, and *Vegf* are up regulated, will transform into hypertrophic chondrocytes and initiate the process of endochondral ossification.^[^ ^22^ ^]^ Macrophages in tissues which polarize into M2 type under the regulation of *Arg1*, *Mcp1*, and *Il10*, promote the process of bone remodeling. Scaffold materials, whether as structural support or delivery system,^[^ ^23^ ^]^ ultimately influence cell functions (migration, differentiation, proliferation, and polarization) by changing genes expression and signal transduction of single cell or cell–cell interaction at the transcriptional level. For example, photo crosslinking gelatin‐methacryloyl (GelMA) loading osteoblast growth peptide (OGP) can target the function of osteoblasts to accelerate the process of osteogenesis in bone repair.^[^ ^24^ ^]^ BMP2 loaded by hydrogel, perichondrium extracellular matrix (PEM), tricalcium phosphate (TCP),^[^ ^25^ ^]^ and poly‐L‐lactic acid (PLLA) electrospinning membrane^[^ ^26^ ^]^ can promote the mineralization of extracellular matrix and osteogenesis.^[^ ^27^ ^]^ Hydrogel scaffolds loaded with platelet‐rich plasma (PRP) target to regulate the function of chondrocytes and promote the formation of cartilage tissues.^[^ ^28^ ^]^ PCL scaffolds loaded with deferoxamine (DFO) promote vascularization and enhance the process of bone remodeling by targeting the regulation of Hif signaling pathway.^[^ ^29^ ^]^ Specific miRNAs or siRNAs are also delivered to regulate the expression of target genes and mediate bone repair stages.^[^ ^30^ ^]^ However, although targeted regulation of single gene, pathway, or protein can achieve the increase of new bone mass, it remains to be studied that the impact of defect area microenvironment induced by biomaterials implantation, including the specific regulatory mechanisms activated by the symbiosis niche to promote regeneration processes and possible emerged side effects to the healing stage. Therefore, RNA‐Seq analysis^[^ ^31^ ^]^ of transcription‐specific activation genes and signal transduction of different scaffold materials in the process of bone regeneration is of great significance for the utilization favorable characteristics of different materials and the regulation of cell behavior in the process of bone regeneration.

**Scheme 1:**
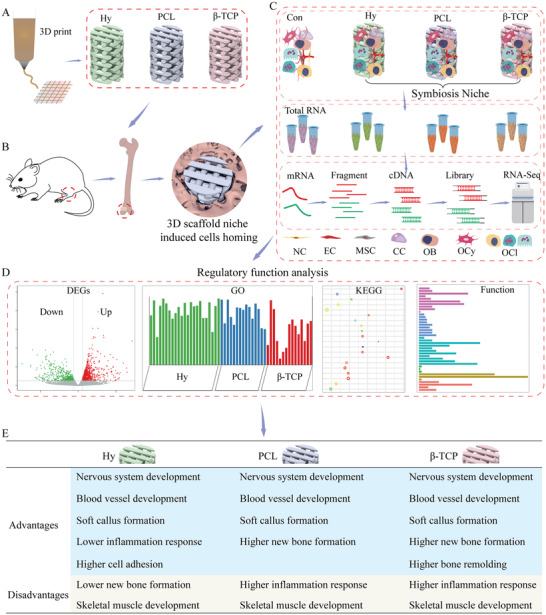
Transcriptome analysis reveals the main regulatory function of hydrogel, PCL, and *β*‐TCP 3D print scaffolds for promoting bone defect healing. A) Fabrication of hydrogel, PCL, and *β*‐TCP scaffolds by 3D printing technology. B) The scaffolds were implanted into a critical‐sized bone defect model at distal femur. C) The regenerate tissues were harvested, and the RNA library were constructed for RNA‐Seq. D) DEGs, GO enrichment, KEGG enrichment, and function classification analysis were carried out to clarify the regulatory function. E) The summary of advantages and disadvantages of hydrogel, PCL, and *β*‐TCP scaffolds during bone healing processes. Hy, hydrogel; PCL, polycaprolactone; *β*‐TCP, *β*‐tricalcium phosphate; BP, biological process; CC, cellular component; MF, molecular function; NC, nerve cell; EC, endothelial cell; MSC, mesenchymal stem cell; CC, cartilage cell; OB, osteoblast; OCy, osteocyte; OCl, osteoclast; DEGs, differentially expressed genes; GO, gene ontology; KEGG, Kyoto Encyclopedia of Genes and Genomes.

Currently, biomaterials used in biomedical engineering of orthopedic mainly include natural polymer materials,^[^ ^5^, ^32^ ^]^ synthetic polymers, and bioceramics, which applied in the repair and remodeling of bone tissue through 3D printing structure design, material composition design, and delivery carrier.^[^ ^19^, ^33^ ^]^ As bio‐inks, natural polymer hydrogel (GelMA), synthetic polymer material (PCL), and bioceramics (*β*‐TCP) were widely used for bone injury repair in the past several decades. In this study, RNA‐Seq was used to analyze the characteristics of symbiosis niche during bone regeneration stage. The biological characteristics of hydrogel, PCL, and *β*‐TCP scaffold materials were obtained through gene function enrichment analysis. The specific regulation mechanism of symbiotic niche provided by the three 3D scaffold biomaterials on cell function and bone regeneration was evaluated. First, we evaluated the biocompatibility of hydrogel, PCL, and *β*‐TCP scaffold materials with bone marrow stem cells (BMSC), HUVEC, RAW264.7, and Schwann cells in vitro. Second, hydrogel, PCL, and *β*‐TCP scaffolds were implanted in critical sized bone defect model and microCT was used to evaluate the formation of new bone. Then the time point of soft callus formation and entochondrostosis initial stage was chosen to detect differentially expressed genes (DEGs) by RNA‐Seq, and biological function, cell composition, molecular function, and signal pathway are enriched to reveal the characteristics of symbiosis niche. Finally, the differences and similarities of the symbiosis niches provided by hydrogel, PCL, and *β*‐TCP scaffold materials during bone repair processes were systematically explained by histomorphology and DEGs function enrichment analysis. The regulation function of symbiosis niche composed of implantation scaffolds, recruitment cells, and newly formed tissues on tissue regeneration process, cell function, and signal transduction are shown (**Scheme** **1**). The potential molecular biological mechanism is provided for the further research and more efficient use of specific scaffold material for bone regeneration. The side effects that may be induced by scaffold during bone regeneration processes are also proposed to notice.

## 2 Results

### 2.1 GelMA Hydrogel, PCL, and *β*‐TCP Scaffolds Appear with Good Biocompatibility In Vitro

**Figure 1:**
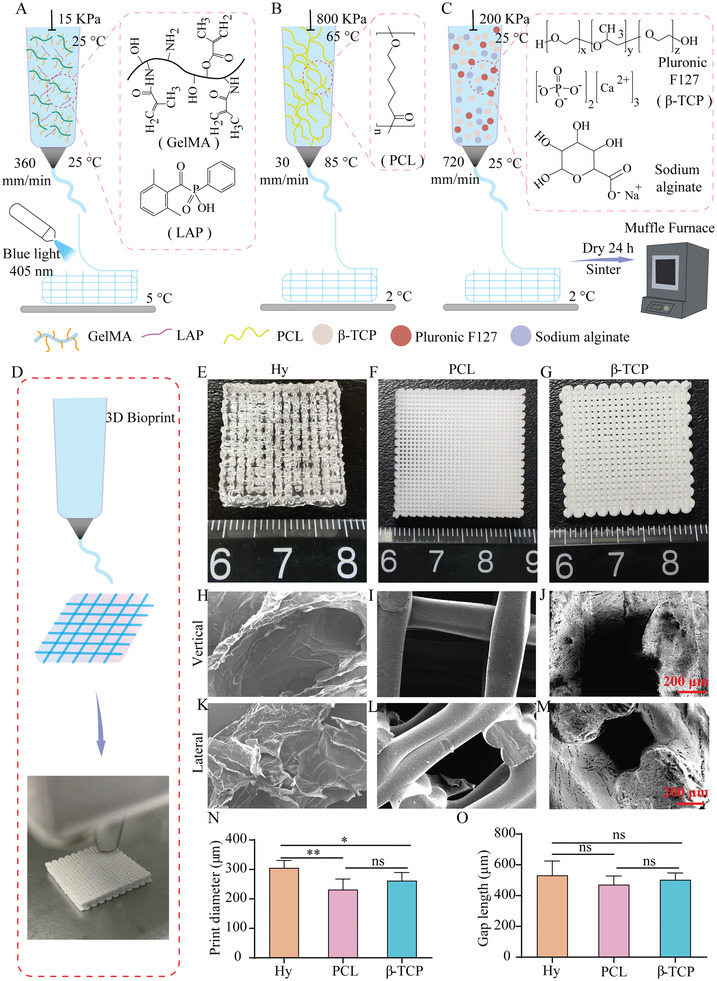
Fabrication and characterization of the GelMA hydrogel, PCL, and *β*‐TCP scaffolds. A–C) The fabrication schematic diagram, relevant parameter, and material components of hydrogel, PCL, and *β*‐TCP scaffolds. D) The general fabrication process of 3D print scaffolds that stacking materials layer by layer. E–G) The scaffolds of hydrogel, PCL, and *β*‐TCP were primary fabricated as a 20 × 20 × 3 mm cube. H–M) SEM images of hydrogel, PCL, and *β*‐TCP scaffolds by vertical and lateral view. N) The statistics of print diameter. O) The statistics of print gap length. Each group contained three replicates and data was analyzed by one‐way ANOVA with Bonferroni test for multiple comparisons (N,O). GelMA, gelatin‐methacryloyl; LAP, lithium phenyl‐2,4,6‐trimethylbenzoyl phosphonate; PCL, polycaprolactone; *β*‐TCP, *β*‐tricalcium phosphate.

To evaluate the function of GelMA hydrogel,^[^ ^32^ ^]^ PCL, and *β*‐TCP 3D scaffolds which promote bone regeneration^[^ ^33^ ^]^ at a critical‐size defect, first, we fabricated a 20 × 20 × 3 mm cuboid scaffold of hydrogel, PCL, and *β*‐TCP scaffolds by proper methods to suit the characteristics of materials (**Figure** **1**A–C). The scaffolds were fabricated by stacking materials layer by layer (Figure 1D). Then, 3 mm diameter cylinder scaffolds were obtained from cuboid scaffolds (Figure 1E–G and Figure S1, Supporting Information). Scanning electron microscope (SEM) images displayed the microstructure of scaffolds surface (Figure 1H–J) and cross‐sectionals (Figure 1K–M). As a result, the hydrogel scaffold's appearance is a porous structure surface, the PCL scaffold's appearance is a smooth surface, and the *β*‐TCP's appearance is a ravines and gullies crisscross surface. Although the same 25G nozzle were used, it was difficult to get the same diameter of single threadlet of hydrogel, PCL, and *β*‐TCP materials (Figure 1N), but the gaps between two threadlet could be controlled in 400–500 µm (Figure 1O).

**Figure 2:**
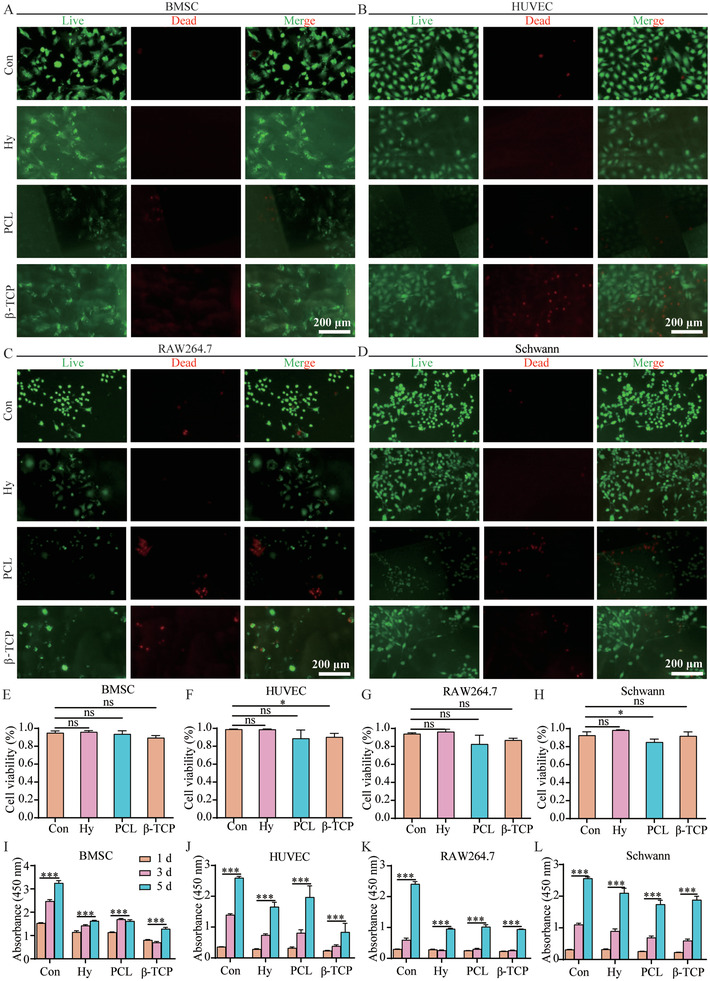
Biocompatibility of the hydrogel, PCL, and *β*‐TCP scaffolds. A–D) Live/Dead staining of BMSC (A), HUVECs (B), RAW264.7 (C), and Schwann (D) cells cultured with no scaffold, hydrogel, PCL, or *β*‐TCP scaffold for 3 days. E–H) The statistics of cell viability of BMSC (E), HUVECs (F), RAW264.7 (G), and Schwann (H) cells cultured with no scaffold, hydrogel, PCL, or *β*‐TCP scaffold for 3 days. I–L) CCK‐8 results of BMSC (I), HUVECs (J), RAW264.7 (K), and Schwann (L) cells proliferation cultured with no scaffold, hydrogel, PCL, or *β*‐TCP scaffold. Each group contained three replicates and data was analyzed by one‐way ANOVA with Bonferroni test for multiple comparisons (E–L). *p < 0.05, *** *p* < 0.001.

During bone regeneration and remodeling, multiple cell types were recruited containing MSCs, macrophages, endothelial cells, and nerve cells to form normal function bone structure.^[^ ^20^, ^21^ ^]^ So, we evaluated the biocompatibility of hydrogel, PCL, and *β*‐TCP scaffolds co‐cultured with BMSC, HUVEC, RAW264.7, and Schwann cells, respectively. The live/dead results showed that hydrogel, PCL, and *β*‐TCP scaffolds groups appeared excellent biocompatibility co‐cultured with all four type cells for 3 d (**Figure** **2**A–D and Figure S2–S5, Supporting Information), although compared to the control group, the PCL scaffold influenced Schwann cells and the *β*‐TCP scaffold influenced HUVEC to a degree (Figure 2E–H). Furthermore, according to the CCK‐8 assay results, there were no significant difference in control and three scaffold groups co‐cultured for 1 day, and the proliferation of BMSC, HUVEC, RAW264.7, and Schwann cells were significant when co‐cultured with scaffolds for 5 days (Figure 2I–L). In a word, as scaffolds, hydrogel, PCL, and *β*‐TCP all appears excellent biocompatibility in vitro.

### 2.2 Scaffolds Accelerate Bone Healing Stage

**Figure 3:**
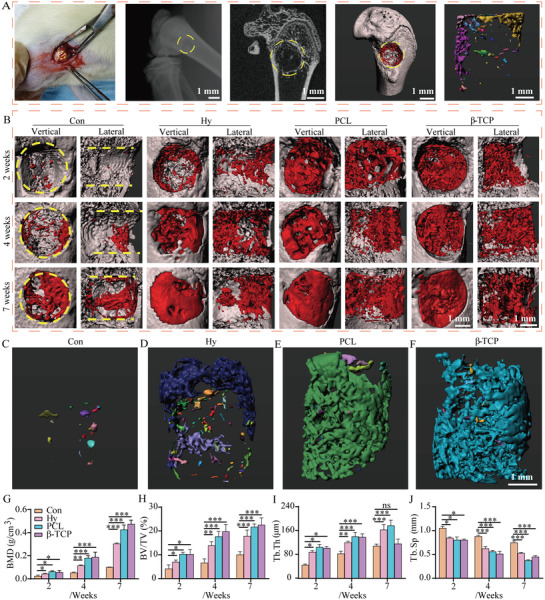
The hydrogel, PCL, and *β*‐TCP scaffolds accelerate bone healing in vivo. A) The critical‐sized bone defect model at distal femur and the implantation of scaffolds, X‐ray to confirm the correct implantation, microCT analysis the formation of new bone, Imaris software was used to differentiate the new bone, and analysis of new bone distribution. B) New bone formation in control, hydrogel, PCL, and *β*‐TCP scaffold groups at 2, 4, and 7 weeks. C–F) The reconstruction of new bone in control, hydrogel, PCL, and *β*‐TCP scaffold groups at 2 weeks. G–J) The statistics of microstructural parameters of new bone, including bone mineral density (BMD), bone tissue volume/total tissue volume (BV/TV), trabecular thickness (Tb.Th), and trabecular separation/spacing (Tb.Sp) at 2, 4, and 7 weeks. Each group contained three replicates and data was analyzed by one‐way ANOVA with Bonferroni test for multiple comparisons (G–J). *p < 0.05, **p < 0.01, ***p < 0.001).

To investigate the function of hydrogel, PCL, and *β*‐TCP scaffold during bone healing, a rat critical‐sized bone defect model was used to evaluate the degree of bone healing at 2, 4, and 7 weeks. In detail, 3D scaffolds were implanted in the distal femur (**Figure** **3**A). After implantation, X ray was used to confirm the scaffold was exactly implanted, then the harvested femurs were scanned by microCT and reconstructed by Imaris software (Figure 3A and Figure S6A–C, Supporting Information). According to the results, only a little new bone could be detected in the inside space of the defect zoon in control group. However, with the implanting of hydrogel, PCL, or *β*‐TCP scaffold, respectively, the volume of new bone was higher than control at the initial 2 weeks and it was more significate when increased until 7 weeks (Figure 3B). The results of reconstruction of new bone at 2 weeks showed the obvious new bone formation in hydrogel, PCL, or *β*‐TCP scaffold groups (Figure 3C–F and Figure S6D,E, and Movies S1–S4, Supporting Information). In addition, the *β*‐TCP scaffold and new bone could be detected at the same time by microCT, which showed the obvious relative location of scaffold and new bones (Figure S7, Supporting Information). Then, the difference of new bone formation was evaluated and quantified by BMD (Figure 3G), BV/TV (Figure 3H), Tb.Th (Figure 3I), and Tb.Sp (Figure 3J), and it was obviously found that the implantation of scaffolds accelerated the bone healing.

**Figure 4:**
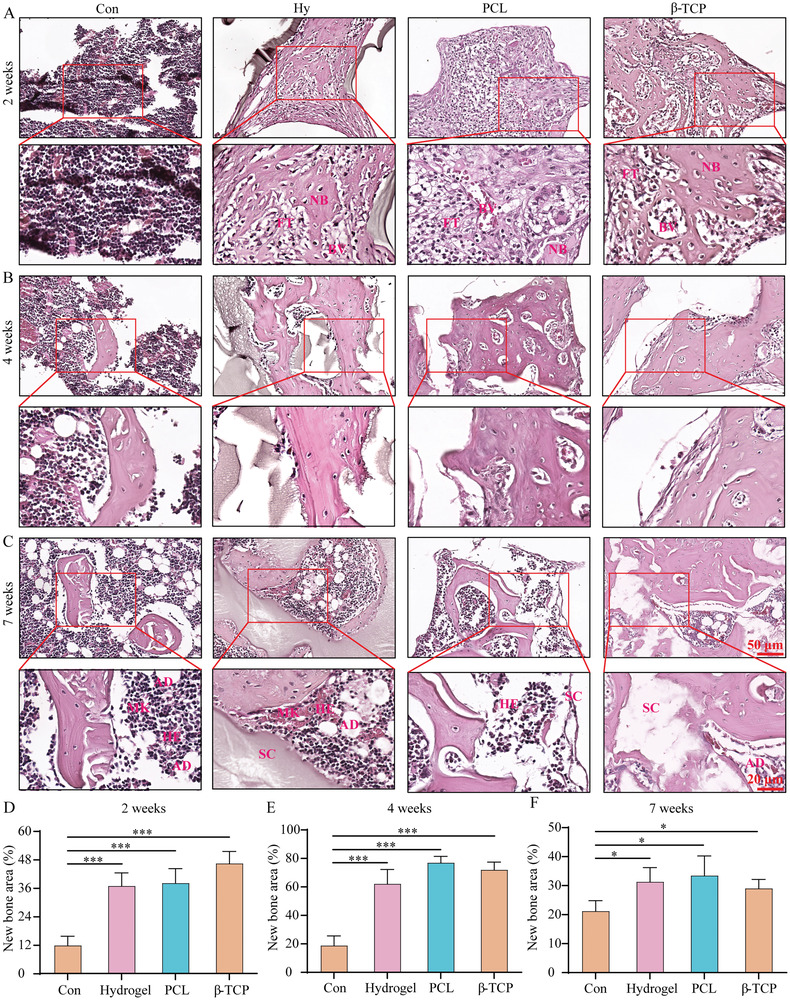
Histomorphology analysis of the new formed tissues in the defect zone. A–C) The new formed tissues in control, hydrogel, PCL, and *β*‐TCP scaffold groups at 2 (A), 4 (B), and 7 weeks (C). D–F) The percent of new formed bone in the regenerate tissues at 2 (D), 4 (E), and 7 weeks (F). Each group contained three replicates and data was analyzed by one‐way ANOVA with Bonferroni test for multiple comparisons (D–F). NB, new bones; BV, blood vessels; FT, fibrous tissue; AD, adipocytes; MK, megakaryocytes; HE, hematopoietic cells; SC, scaffold. *p < 0.05, ***p < 0.001.

In addition, histological analysis of HE staining showed that regenerated tissues initially formed around scaffolds in hydrogel, PCL, and *β*‐TCP groups, but without scaffold, only a little spot volume tissues formed (**Figure** **4**A) at 2 weeks. The staining results revealed that the implantation of scaffolds promoted the formation of ossification centers, new bones, blood vessels, and fibrous tissue in the soft callus (Figure 4A). At 4 weeks, the soft callus was almost replaced by hard callus in scaffolds implantation groups (Figure 4B), but there were both soft callus and hard callus and the volume was less than scaffold groups in the control group (Figure 4B). At 7 weeks, the remodeling stage has been activated in scaffolds group, but there was still a little new bone formed in the control group (Figure 4C). And the morphology of regenerated tissues in control group at 7 weeks was more likely the healing stage of implantation groups at 4 weeks (Figure 4C). During bone defect healing, the main process in the defect zone was endochondral osteogenesis like long bone development. So, the formation of soft callus after inflammatory stage was important for further hard callus formation and bone remodeling processes. Without scaffold, the emerging of ossification was less than scaffolds implantation groups because of the limitation of soft callus formation (Figure 4A–C), and only little ossification spots can be found (Figure S6D,E, Supporting Information) at 2 weeks. However, there were more ossification spots and the spots would form linear bone structure in scaffolds′ implantation groups (Figure S6D,E, Supporting Information). But it was less known about the transcriptome mechanism of signal transduction and regulatory network that the scaffold implantation to promote the formation of soft callus and ossification, and what was the unique role of hydrogel, PCL, or *β*‐TCP scaffold during these processes.

**Figure 5:**
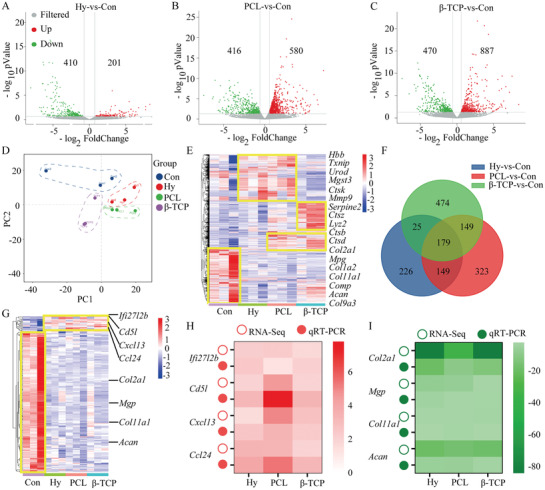
Transcriptome analysis of the scaffolds function to accelerate bone healing. A–C) The DEGs in hydrogel (A), PCL (B), and *β*‐TCP scaffold (C) groups compared with control group. D) PCA analysis of all samples. E) Heatmap analysis of DEGs. F) Venn diagrams showing the number of DEGs in hydrogel, PCL, and *β*‐TCP scaffold groups. G) Heatmap showing the DEGs which all up‐regulated or down‐regulated in hydrogel, PCL, and *β*‐TCP scaffold groups. H) Validation of transcriptome data (up‐regulated DEGs) by quantitative reverse transcription‐polymerase chain reaction (RT‐qPCR). I) Validation of transcriptome data (down‐regulated DEGs) by RT‐qPCR. Each group contained three replicates and DEGs were presented which FoldChange > 2 or FoldChange < 0.5.

To well know the role of hydrogel, PCL, and *β*‐TCP scaffold to accelerate the endochondral osteogenesis process at transcriptome level, expressed genes in control group, hydrogel, PCL, and *β*‐TCP groups at 2 weeks, were obtained by RNA‐Seq. Then the analysis of DEGs was applied and there were 201 DEGs up‐regulated and 410 DEGs down‐regulated in hydrogel group compared with the control group (**Figure** **5**A), there were 580 DEGs up‐regulated and 416 DEGs down‐regulated in PCL group compared with the control group (Figure 5B), and there were 887 DEGs up‐regulated and 470 DEGs down‐regulated in *β*‐TCP group compared with the control group (Figure 5C). The statistical analysis of the samples in four groups was accomplished by principal component analysis (PCA), and the results show that the transcriptome data can be used for further analysis (Figure 5D).The heatmap revealed the expression pattern of DEGs (Figure 5E), and hydrogel and PCL groups had the similar expression pattern comparing with the control, but the *β*‐TCP group had a special pattern compared to the control, hydrogel, and PCL groups, which indicated that although different scaffolds could all promote bone healing process, the innate mechanism might be utterly different. Then the DEGs was enriched in hydrogel, PCL, and *β*‐TCP groups comparing with the control, and 179 DEGs were included in all three groups, which might be induced by the implantation of scaffolds (Figure 5F). Further analysis of the 179 DEGs revealed that 18 DEGs were up‐regulated and 161 DEGs were down‐regulated (Figure 5G). To validate the DEGs identified by transcriptome analysis, up and down‐regulated DEGs were selected for RT‐qPCR analysis (Figure 5H,I). The primer sequences are listed in Table S1, Supporting Information.

### 2.3 DEGs Comparison Revealed a Scaffold Characteristic

**Figure 6:**
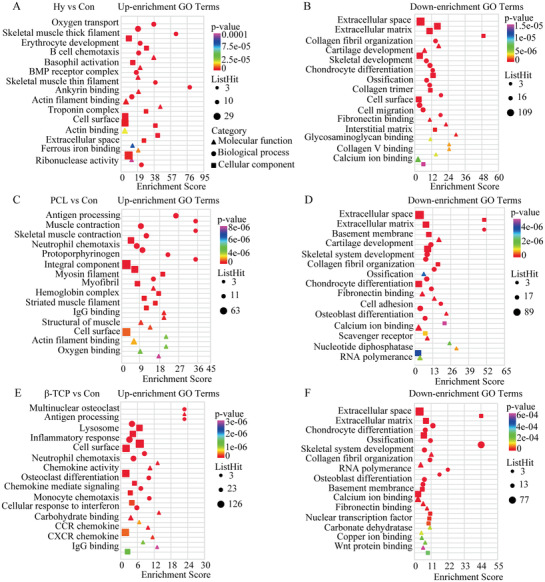
GO enrichment analysis of DEGs in scaffold group compared with control group. A) The top 15 up enrichment GO terms in hydrogel group. B) The top 15 down enrichment GO terms in hydrogel group. C) The top 15 up enrichment GO terms in PCL group. D) The top 15 down enrichment GO terms in PCL group. E) The top 15 up enrichment GO terms in *β*‐TCP group. F) The top 15 down enrichment GO terms in *β*‐TCP group.

**Figure 7:**
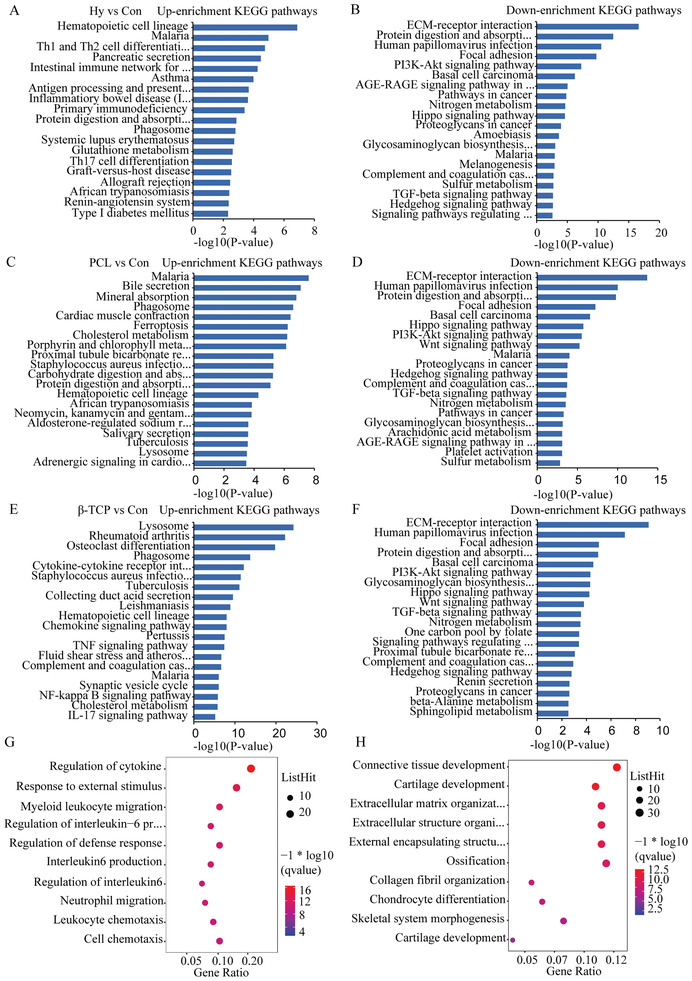
KEGG enrichment analysis of DEGs scaffold group compared with control group. A) The top 15 up enrichment KEGG pathways in hydrogel group. B) The top 15 down enrichment KEGG pathways in hydrogel group. C) The top 15 up enrichment KEGG pathways in PCL group. D) The top 15 down enrichment KEGG pathways in PCL group. E) The top 15 up enrichment KEGG pathways in *β*‐TCP group. F) The top 15 down enrichment KEGG pathways in *β*‐TCP group. G) The top 10 up enrichment GO terms in least two groups of hydrogel, PCL, and *β*‐TCP group compared with control group. H) The top 10 down enrichment GO terms in least two groups of hydrogel, PCL, and *β*‐TCP group compared with control group.

Although bone healing was promoted after scaffolds implantation at histological level (Figure 3), the function of DEGs and the relationship between DEGs and bone healing were not clear. In this study, GO enrichment was used to analyze the function of DEGs at biological processes, cellular components, and molecular function level. Comparing to the control group, oxygen transport, a skeletal muscle thick filament, and erythrocyte development were up‐regulated and extracellular space, extracellular matrix, and collagen fibril organization were down‐regulated in the hydrogel group (**Figure** **6**A,B), antigen processing, muscle contraction, and skeletal muscle contraction were up‐regulated and extracellular space, extracellular matrix, and basement membrane were down‐regulated in the PCL group (Figure 6C,D), and GO terms of multinuclear osteoclast, antigen processing, and lysosome were up‐regulated and extracellular space, extracellular matrix, and chondrocyte differentiation were down‐regulated in the *β*‐TCP scaffold group (Figure 6E,F). In addition, KEGG pathway enrichment analysis was also used to reveal the functions of DEGs. Comparing to the control group, hematopoietic cell lineage, malaria, and T cells differentiation pathways were up‐regulated and EMC‐receptor interaction, protein digestion, and human papillomavirus infection were down‐regulated in the hydrogel group (**Figure** **7**A,B), malaria, bile secretion, and mineral absorption were up‐regulated, and EMC‐receptor interaction, human papillomavirus, and protein digestion were down‐regulated in the PCL group (Figure 7C,D), and lysosome, rheumatoid arthritis, and osteoclast differentiation pathways were up‐regulated and EMC‐receptor interaction, human papillomavirus, and focal adhesion were down‐regulated in the *β*‐TCP group (Figure 7E,F). Then, the DEGs which contained at least in two groups, which indicated a common characteristic of scaffolds, were analyzed by GO enrichment. The up enrichment GO terms were the regulation of cytokine, defense response, IL6 pathway, neutrophil migration, and cell chemotaxis, and the down enrichment GO terms were tissue development, cartilage development, extracellular space, and chondrocyte differentiation (Figure 7G,H).

**Figure 8:**
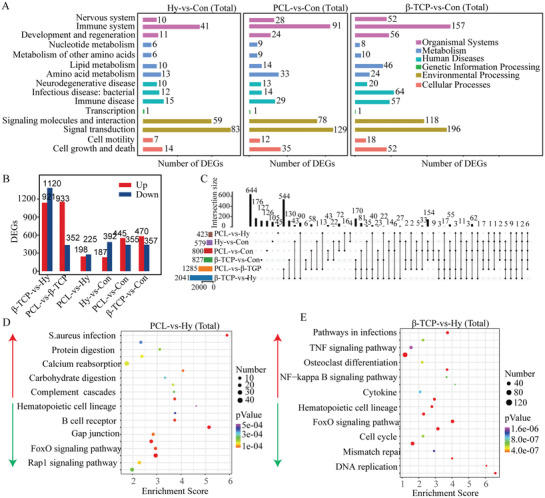
Function analysis of DEGs in hydrogel, PCL, and *β*‐TCP scaffold groups. A) Comparing to control group, the distribution of DEGs in hydrogel, PCL, and *β*‐TCP scaffold groups. B) DEGs in hydrogel, PCL, or *β*‐TCP group compared with other groups. C) The DEGs shared by different groups. D) KEGG analysis of DEGs in PCL groups compared with hydrogel group (up‐enrichment and down‐enrichment). E) KEGG analysis of DEGs in *β*‐TCP groups compared with hydrogel group.

The above GO and KEGG results matched with the histomorphology results. In the control group, soft callus started to form at 2 weeks, as a result, the cartilage development, collagen formation, and chondrocyte differentiation related functions were up‐enrichment (Hippo, Wnt, TGF*β*, and Hedgehog signaling pathways). However, the entochondrostosis process has been started and hard callus formed in hydrogel, PCL, and *β*‐TCP scaffold groups, so the oxygen transport, erythrocyte, cell surface, cell chemokine, absorption, and metabolism related GO terms and KEGG pathways were up‐enriched (Figures 6 and 7). In summary, the main function of hydrogel, PCL, and *β*‐TCP scaffolds to promote bone healing were embodied in the nervous system, immune system, development and regeneration, metabolism, and signal transduction (**Figure** **8**A). The *β*‐TCP scaffold group contained more DEGs and more obvious change in the function terms.

Further, the DEGs among three scaffolds groups were also analyzed besides comparing to the control group, respectively. The results shown that there were specific DEGs in hydrogel, PCL, or *β*‐TCP scaffolds comparing to other two groups (Figure 8B), and a part of DEGs were shared by two or all three scaffold groups (Figure 8C). To well know the difference of the regulation response induced by hydrogel, PCL, or *β*‐TCP scaffold, GO enrichment analysis was applied. The results show that PCL and *β*‐TCP promoted calcium reabsorption and an osteoclast differentiation function to accelerate bone regeneration, but the more obvious activation of immune related pathways were also induced compared with hydrogel. The main function of hydrogel was the regulation of cellular processes like cell differentiation (hematopoietic cell lineage) and cell‐cycle control (FoxO signaling pathway, cell cycle, and DNA replication) (Figure 8D,E). In addition, the *β*‐TCP scaffold group appeared with less cell cycle and a mineral absorption related pathway enrichment and more innate immune related pathways enrichment (Figure S8, Supporting Information). On the other hand, although the symbiosis niche comprised of scaffolds, homing cells, and regenerated tissues promote the bone healing processes, it was non‐ignorable that the inflammation response signal pathways were up‐enriched in all scaffold groups (Figure 7G), but the response degree was lowest in the hydrogel group and highest in *β*‐TCP scaffold group (Figure 8D,E and Figure S8, Supporting Information).

**Figure 9:**
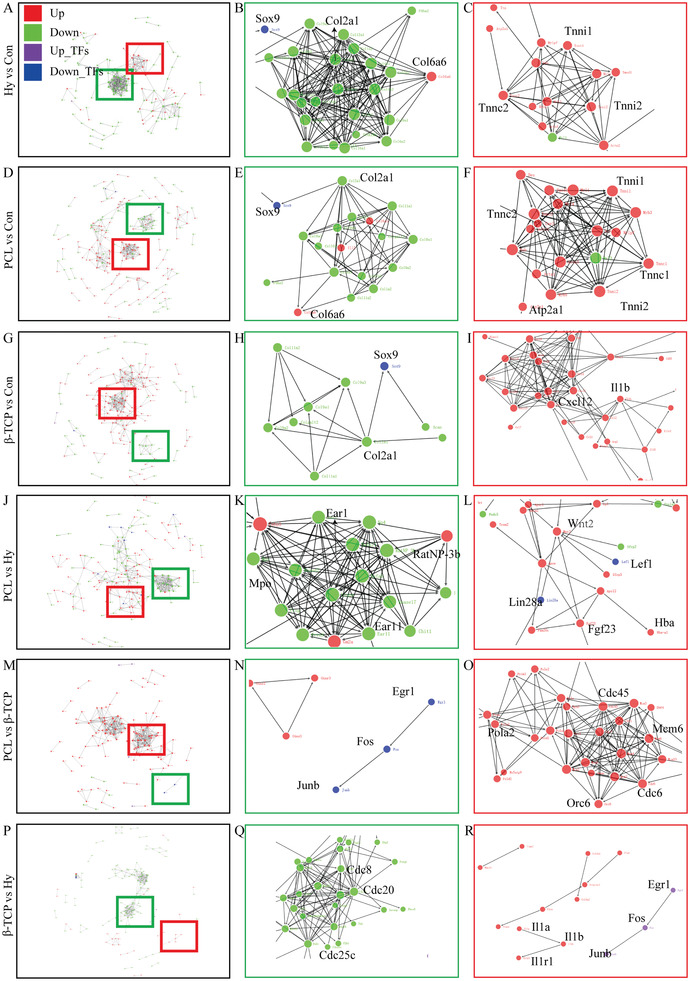
Protein–protein interaction network (PPI) analysis. A–C) DEGs in hydrogel group compared with control group. D–F) DEGs in PCL group compared with control group. G–I) DEGs in *β*‐TCP group compared with control group. J–L) DEGs in PCL group compared with hydrogel group. M–O) DEGs in PCL group compared with *β*‐TCP group. P–R) DEGs in *β*‐TCP group compared with hydrogel group. The red color represents up‐regulated proteins, green represents down‐regulated proteins, purple represents up‐regulated transcriptome factors, and blue represents down‐regulated transcriptome factors. The red color represents up‐regulated proteins, green represents down‐regulated proteins, purple represents up‐regulated transcriptome factors, and blue represents down‐regulated transcriptome factors.

In addition, the protein–protein interaction network (PPI) analysis was also preformed to reveal the proteins functions encoded by DEGs in different groups (**Figure** **9**A–R, and sPPI 1–6, Supporting Information). Confirmed with the GO and KEGG enrichment, the main down regulated network was collagen and extracellular space related (Figure 9B,E,H,K) in all scaffold groups compared with the control group. The up regulated network in hydrogel and PCL group were a troponin related network (Tnni1, Tnni2, Tnnc1, and Tnnc2), but the main change protein network in the *β*‐TCP group was immune (Il1b) and chemokines (Cxcl12) related. In addition, the PPI analysis were also performed among hydrogel, PCL, and *β*‐TCP group, the results show that cell division cycle protein (Cdc protein family) were up‐enriched in the hydrogel and PCL group (Figure 9O,Q). The immune related proteins (Il1a, Il1b, and Il1r1) and cell proliferation, differentiation, and transformation related proteins (Erg1, Fos, and Junb) were up enriched in the *β*‐TCP group which may related to the obvious immune response and osteoclast differentiation induced by *β*‐TCP during bone healing (Figure 9N,R).

**Figure 10:**
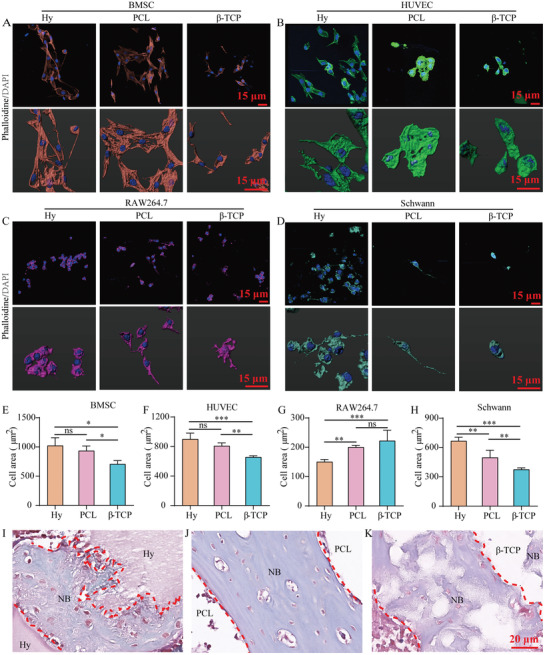
The regulatory role of scaffolds to influence cells or tissues in symbiosis niche in vitro and in vivo. A) The cellular morphology of BMSC in hydrogel, PCL, or *β*‐TCP scaffold niche. B) HUVEC. C) RAW264.7. D) Schwann cells. E) The statistic of BMSC cell area in hydrogel, PCL, or *β*‐TCP scaffold niche. F) HUVEC. G) RAW264.7. H) Schwann cells. I) The symbiosis niche of hydrogel scaffold and new tissue. J) PCL scaffold. K) *β*‐TCP scaffold. The red dashed line indicates the interface of scaffold and new tissue. NB: new bone. ns: no significance. Each group contained three replicates and data was analyzed by one‐way ANOVA with Bonferroni test for multiple comparisons (E–H). *: *p* < 0.05, **: *p* < 0.01, ***: *p* < 0.001.

To further verify the regulation function of hydrogel, PCL, and *β*‐TCP to influence the symbiosis niche, which was comprised of scaffold, cells, and regenerated tissues, we co‐cultured the BMSC, HUVEC, RAW264.7, and Schwann cells with hydrogel, PCL, or *β*‐TCP scaffolds in vitro (**Figure** **10**A–D). The cell area evaluation (Figure 10E–H) and biocompatibility (Figure 2) results revealed that hydrogel and PCL promote BMSC, HUVEC, and Schwann cells growth, but *β*‐TCP has an obvious role to regulate RAW264.7 cells. Histomorphology analysis had shown that there was significant difference of interface between scaffold and new tissues after 7 weeks of implantation. Although all hydrogel, PCL, or *β*‐TCP scaffold could accelerate the bone healing processes, the hydrogel scaffold could be absorbed and the increase of interface area promotes cell adhesion and gap junction (Figure 10I), the PCL scaffold limits new bone formation in the gap and maintains the existence of the interface all the time (Figure 10J), and the *β*‐TCP scaffold could promote new bone remodeling by regulating macrophage function and osteoclast differentiation (Figure 10K).

## 3 Discussion

In this study, the hydrogel, PCL, and *β*‐TCP 3D printed scaffolds are implanted into a critical‐sized defect model, and the niche provided by scaffolds promotes cell recruitment, extracellular matrix adhesion, and new tissue formation. The symbiosis niche formed by scaffolds, cells, and regenerated tissues accelerates bone healing processes by influencing genes expression, signal transduction, and cellular function in the microenvironment. The results showed that the implantation of the three scaffolds could accelerate the process of bone repair, but the characteristics of symbiosis niche including gene expression, regulatory network, cell behavior, and histomorphology are different. The hydrogel scaffold promotes cell adhesion and proliferation, including regenerated bone tissue cells, B cells, and macrophages. The PCL scaffold is beneficial to cell proliferation and differentiation. The *β*‐TCP scaffold improves the processes of osteoclast differentiation and bone remodeling. Second, although all the three scaffolds perform excellent biocompatibility in vitro, inflammatory responses are activated in all the three scaffolds group, with the formation of symbiosis niche, among which *β*‐TCP scaffold caused the highest inflammatory response, followed by PCL and hydrogel. In addition, the enrichment of pathways related to muscle development and formation also suggests that while scaffold materials promote bone healing, they may also cause abnormal proliferation of muscle tissue at the bone–muscle interface. Our results suggest that other natural and synthetic materials of the 3D printed scaffold may also have the universal function referred in the DEGs enrichment analysis, to promote the bone healing processes. But the inflammation response and muscle regeneration induced by scaffolds in the symbiosis niche microenvironment are potential side effects that need to be noticed and the specific characteristics of other materials may exist and can be used for improving the symbiosis niche. For further studies on biomaterials for bone healing, the specific genes and signal pathways activated by scaffolds should be considered, and combined biomaterials may be designed to make full use of the advantages of different materials and avoid the side effect of materials, to improve biological safety and effectively and bone healing processes.

The inflammatory response activated in the symbiosis niche also inescapably appears and exists after the inflammation stage. In general, the inflammatory response is triggered at the early stage of hematoma formation during bone regeneration. The cells of neutrophils, M1 macrophages, and CD8^+^ T cells release pro‐inflammatory factors IL1*β*, IL6, and TNF*α*. In the subsequent stage of granulation tissue formation, macrophages secrete anti‐inflammatory factors IL10, and growth factors to inhibit inflammatory response and promote the formation of cartilage callus.^[^ ^21d^ ^]^ In our results, after the implantation of hydrogel, PCL, and *β*‐TCP scaffolds, significant ossification centers were found in soft callus and hard callus that began to form at 2 weeks, indicating that bone healing had undergone the phase of inflammatory response and early granulation tissue formation. However, GO and KEGG analysis of DEGs in transcriptome data show that a large number of immune‐related pathways were still activated and the inflammatory response continued (Figures 6 and 7). Due to critical bone defect not being self‐healing, the scaffold material provides a good microenvironment for tissue regeneration and the formation of symbiosis niche to further improve bone healing progresses. However, even the scaffolds appear with good biocompatibility in vitro, but as a foreign body, a degree of immune response can also be activated that affect bone healing processes, by inducing coagulation cascade and activating the complement system.^[^ ^34^ ^]^ Studies have shown that although inflammation is beneficial to the formation of early granulation tissue, it has an inhibitory effect on the osteogenic differentiation of MSC in vitro,^[^ ^35^ ^]^ and bone regeneration is promoted in RAG^−/−^ mice which lack adaptive immunity.^[^ ^36^ ^]^ So, persistent inflammation response activation may not be conducive to bone healing, and the osteogenesis effect is better than no implant group in the symbiosis niche, probably due to the existence of the scaffold material that provides more endothelial progenitor cells and MSC adhesion sites, generating more granulation tissue at early age. At the same time, it also provides adhesion sites for immune cells and as a foreign body, making the inflammatory response continuously activated.^[^ ^37^ ^]^ Comparing the inflammatory response induced by hydrogel, PCL, and *β*‐TCP scaffolds, that hydrogel causes moderate inflammatory response and is followed by PCL scaffolds. However, *β*‐TCP scaffolds cause the most severe inflammatory response and a large number of complement system genes are activated. These results suggest that when designing scaffold materials, attention should be paid to the surface of materials to increase MSC adhesion while minimizing excessive adhesion of platelets and immune cells to inhibit persistent inflammatory responses. In addition, excessive inflammatory reactions should be avoided, which is conducive to improving the feasibility of using scaffold materials for non‐healthy patients (such as cancer, osteoporosis, and diabetes) in clinic.

As a polymer, the hydrogel has obvious advantages in transcriptional regulation compared with PCL and *β*‐TCP scaffolds in bone healing. In addition to the mild inflammatory responses, the GO function and KEGG pathway related to oxygen transport and red blood cell development were significantly up‐regulated. Combined with histological results, it was found that the porous network structure of the hydrogel increased the interface between cells and the scaffold, which would contribute to the formation of the initial granulation tissue. Further, GO and KEGG enrichment reveals that the function of B cells and leukocyte proliferation and adhesion related pathways are also of significant enrichment. However, the inflammatory response in the hydrogel implantation group is a minimum. In the case of low inflammatory response, whether the adhesion of B cells and leukocyte in the regenerated tissue region is beneficial to the bone regeneration process still needs further research. Due to the convenient design of 3D structure (such as microspheres, nanoparticles, and fibers), good absorbability, controllable degradation rate and excellent delivery function of hydrogels,^[^ ^32^, ^38^ ^]^ mechanisms of immune cell types, and related signaling pathways involved in this study can be further studied and using hydrogel scaffold to provide a symbiosis niche and improve bone regeneration by targeting inflammatory response. In addition, it is worth noting that the natural bone healing phases include inflammatory response, granulation tissue, soft callus, hard callus, and bone remodeling,^[^ ^19^, ^20^, ^21^ ^]^ but the symbiosis niche provided by scaffold will shorten the duration of each phase and accelerate the whole bone healing process. So, if the scaffold materials are used as delivery system and target a specific regeneration stage to achieve precise control of the healing stage, the release time point and rate should match the changed processes not the natural processes. However, as a powerful tool for clinical treatment of bone defects, further research still needs to improve the characteristics of hydrogel in degradation control, intelligent release, and mechanical strength.

A combined design of bio‐scaffold might be considered to utilize material characteristic and achieve better bone healing. The transcriptome results show that there are specific DEGs and signal pathways up or down regulated in hydrogel, PCL, and *β*‐TCP scaffolds group, besides the improvement of bone healing. Because of the better mechanical support function, there are more newly formed bone mass in the PCL and *β*‐TCP scaffolds group compared with the hydrogel group, although they have obvious inflammatory response. The composite materials have been attempted as a delivery system to inhibit inflammation and promote bone repair.^[^ ^39^ ^]^ based on the biological characteristic of scaffolds including degradation rate, porosity, release curve, and other advantages^[^ ^32^ ^]^ and the transcriptome level response activated in symbiosis niche. A composite scaffold material can be designed that uses a synthetic polymer material to enhance mechanical strength, bioceramics to promote bone remodeling, and hydrogel to reduce inflammatory response. Thus, a simple scaffold material not as a delivery system, can achieve phased regulation of bone healing processes by a safe and efficient way.

The potential side effects also need to be paid attention to, which may lead to the abnormal proliferation of skeletal muscle. As a symbiosis niche, the scaffold not only recruits leukocytes, erythrocyte, and MSCs, but also other cells which have an interaction with the defect area. The results of GO and KEGG enrichment reveal that proliferation, differentiation, and adhesion related pathways of muscle cells were also significantly up‐regulated, indicating that a scaffold material promotes proliferation and adhesion of surrounding muscle tissue at the interface between bone defect area which easily leads to tissue adhesion in the regeneration process. Therefore, anti‐adhesion factors should be considered in the surface during the designing of scaffold materials. The interface between bone tissue and surrounding muscle tissue (such as changing the physical and chemical properties of the material surface^[^ ^40^ ^]^ or adding factors that inhibit the growth of muscle cells) should be considered to avoid the occurrence of tissue adhesion during tissue regeneration.

## 4 Conclusion

Currently, hydrogel, PCL, and *β*‐TCP have been extensively researched and used as scaffold materials for regenerative medicine and have great potential in the repair of bone and cartilage. The implantation of scaffold provides a niche to recruitment cells and tissue formation forming a symbiosis microenvironment. The transcriptome function analysis has board implications for regenerative medicine providing insight into the functions, regulation, and interactions by the scaffold and cells. It identifies those directly affected by different scaffold materials and distinguishes the effective signaling pathways to improve bone healing and potential side effects induced by scaffolds. These results define specific genes and signal pathways activated in a symbiosis niche and provide optional targets to improve bone healing. Further studies need to verify the regulation mechanism and possible side effects of a scaffold material after implantation by molecular biological methods to promote the process of forming engineered products and applying in clinic. The characteristics of different materials that induce transcriptome response should be considered in further research and fabrication of scaffold to improve the safety and efficiency of tissue regeneration.

## 5 Experimental Section

### Fabrication of 3D Print Scaffolds

In this study, GelMA, PCL, and*β*‐TCP were used for synthesis of 3D scaffolds. The scaffolds were manufactured using an extrusion‐based 3D printer (EFL‐BP6601, EFL, China). Briefly, 5% GelMA was dissolved into ddH2O with lithium phenyl (2,4,6‐trimethylbenzoyl) phosphinate (LAP, MACKLIN, China) (0.0025 g mL^−1^) at 37 °C for 30 min, and the 25G nozzle was heated to 25 °C with a pressure of 15 KPa. The speed was 360 mm min^−1^, the temperate of the baseplate was 4 °C and during printing, 405 nm light was used to crosslink scaffold. The PCL was loaded into the material chamber and heated to 65 °C and the yield stress was measured as 800 KPa to extrude the liquid PCL, and the 25G nozzle was heated to 85 °C. The printing speed was 30 mm min^−1^. The injectable *β*‐TCP bio‐ink was prepared by the method as previously reported,^[^ ^41^ ^]^ briefly, 44g *β*‐TCP powders (MACKLIN, China), 18g sodium alginate suspension (10 wt%) (MACKLIN, China), 5 g Pluronics F‐127 solution (20 wt%) (Aladdin, China), and 6 mL ddH2O were mixed by milling. Then *β*‐TCP bio‐ink was placed into printing tubes with a pressure of 200 KPa, and 25 G nozzle was used with the printing speed of 720 mm min^−1^, and the temperate of the baseplate was 2 °C. The printed *β*‐TCP scaffolds were dried at room temperature for 24 h and sintered at 1100 °C for 2 h at a heating rate of 10 °C min^−1^. The fabrication of GelMA, PCL, and *β*‐TCP scaffolds with the standard of that the thickness of each piece was 0.18–0.22 mm, and the gap of scaffolds were 400–500 µm.

### Characterization of the Scaffolds

The 3 mm column scaffolds were obtained from printed cube scaffolds. The macropores and microstructure of the pore walls were characterized by scanning electron microscopy (SEM, Sirion 200, FEI, USA).

### Biocompatibility Test

The rat BMSC and mouse leukemic monocyte/macrophage cell line RAW264.7 cells were kindly provided by Tao Wang (Department of Orthopaedics, Shanghai Key Laboratory for Prevention and Treatment of Bone and Joint Diseases, Shanghai Institute of Traumatology and Orthopaedics, Ruijin Hospital, Shanghai Jiao Tong University School of Medicine). The human umbilical vein endothelial cells (HUVEC)^[^ ^42^ ^]^ and Schwann^[^ ^43^ ^]^ cells were kindly provided by Juan Wang (Department of Orthopaedics, Shanghai Key Laboratory for Prevention and Treatment of Bone and Joint Diseases, Shanghai Institute of Traumatology and Orthopaedics, Ruijin Hospital, Shanghai Jiao Tong University School of Medicine). The four type cells were chosen to investigate the biocompatibility. These cells were co‐cultured with these scaffolds, respectively, in a 24‐well cell culture plate (10000 cells per well). The *α*‐modified eagle medium (*α*MEM, Gibco, USA) with 10% fetal bovine serum (FBS, Gibco, USA) and 1% penicillin‐streptomycin solution (HyClone, USA) was used to culture cells at 37 °C with 5% CO_2_.

A cell counting kit‐8 (CCK‐8, YEASEN, China) was employed to analyze the proliferation and cytotoxicity of BMSC, RAW264.7, HUVEC, and Schwann cells on three scaffolds, which was performed according to the protocol provided by the product's company. Briefly, the test reagent was mixed with *α*MEM on day 1, 3, and 5, and incubated for 2 h. Then the absorbance was quantified (iMark Microplate Reader, BIO‐RAD, USA) at 450 nm.

A Live/Dead Cell kit (YEASEN, China) was applied to assess the viability of cells on three scaffolds. After incubation, the fluorescence images were acquired by a fluorescence microscope (Nikon Corporation, Japan). According to the manufacturer's protocol, calcein‐AM (green fluorescence) represents viable cells and propidium iodide (PI, red fluorescence) represents dead cells; these were used to assess cell viability. The cells were at the same passage number for in vitro studies.

### Animal Models

Rat distal femoral bone critical size defect model was established for in vivo bone repair assessment by the method as previously reported.^[^ ^44^ ^]^ Briefly, 8 weeks old Sprague Dawley (SD) rat (300 ± 50 g) were anesthetized by 2.5% pentobarbital sodium (Sangon, China), and the lateral longitudinal incision of the distal femur is taken to expose the lateral femoral condyle, and a 3 mm electric drill was used to penetrate the bone cortex. Then Hy, PCL, and *β*‐TCP scaffolds were implanted into the defect site, and the control group didn't implant on the scaffold. Every group contains 18 rats, and the authors harvested the rat femurs of the four groups at 2, 4, and 7 weeks after implantation (each group contained 6 samples, *n* = 6). All animal experiments were approved by the Animal Ethics Committee, Ruijin Hospital, Shanghai (SYXK 2018‐0027).

### Histological Analysis

The femurs contained scaffold and control group were harvested and fixed with 4% paraformaldehyde (PFA, Sangon, China). Then microCT (SKYSCAN 1172, Belgium) and X‐ray (Faxitron x‐ray, USA) were used to evaluate the damage repair. The software Imaris 9.2 (Oxford Instruments, UK) was used to reconstruct the 3D bone model. Specifically, the surface function section was used to reconstruct a space structure based on microCT scanning image. The boundary of different parts was the recognition and surface of every respective part according to the manufacturer's instructions (https://imaris.oxinst.com). After that, samples were placed in 10% ethylene diamine tetraacetic acid (EDTA, Sangon, China) for decalcification before being embedded in paraffin. Then, 5 µm thick sections were used for hematoxylin and eosin (HE) staining analysis with the methods previously reported.^[^ ^29^, ^44^ ^]^

### Transcriptome Analysis

The symbiosis niches were used for transcriptome analysis at 2 weeks. Briefly, samples of newly formed tissues in the control group and symbiosis niches of Hy, PCL, and *β*‐TCP scaffold groups were grinded under liquid nitrogen. The total RNA was isolated with the Trizol (Vazyme, China) according to the manufacturer's instructions. The concentration of RNA was measured with a NanoDrop 2000c (Themo, USA) and its quality was measured with an Agilent 4100 Bioanalyzer (Agilent, USA). The RNA‐seq libraries were constructed using VAHTS Universal V8 RNA‐seq Library Prep Kit for Illumina (Vazyme, China), and the libraries were deep sequenced using paired‐end sequencing with the Illumina NovaSeq 6000 (Illumina, USA), according to the manufacturer's protocol. Mapping and enrichment analysis were performed as previously described.^[^ ^45^ ^]^ The transcriptome sequencing was conducted by OE biotech Co Ltd (Shanghai, China). Cleaning reads were obtained using Trimmomatic and mapped to reference genome using hisat2. FPKM (fragments per kilobase of exon per million reads mapped) value of each gene was calculated using cufflinks. The DEGs, GO (gene ontology), and KEGG (Kyoto Encyclopedia of Genes and Genomes) enrichment analysis were performed using R software. *p* value < 0.05 and FoldChange > 2 or FoldChange < 0.5 was set as the threshold for significantly differential expression or differential enrichment.

### Quantitative Reverse Transcription‐Polymerase Chain Reaction (RT‐qPCR)

The cDNA synthesis was performed using HiScript SuperMix for qPCR kit (Vazyme, China), RT‐qPCR was performed using a Roche 480 real‐time PCR detection system (Roche, Switzerland). Results were normalized to GAPDH and were analyzed using the 2^−△△Ct^ method. The primer sequences are listed in Table S1, Supporting Information.

### Statistical Analysis

The pre‐processing of transcriptome data was performed using R according to the standard threshold value. Values were expressed as the means ± SEM, and the difference between groups was analyzed by one‐way ANOVA with Bonferroni test for multiple comparisons. At least three independent samples were contained in each group for each statistical analysis. Column statistics were performed on datasets to check for normal distribution. Statistical analyses were performed using Excel (Microsoft, USA), SPSS (IBM Corp, USA), GraphPad Prism 9 (GraphPad Software, USA), and R software.

## Conflict of Interest

The authors declare no conflict of interest.

## Supporting information

Supporting Information

Supplemental Video 1

Supplemental Video 2

Supplemental Video 3

Supplemental Video 4

Supplemental Video 5

Supplemental Video 6

Supplemental Video 7

Supplemental Video 8

Supplemental Video 9

Supplemental Video 10

## Data Availability

The data that support the findings of this study are available in the supplementary material of this article.
