# Supplementary material for: Transcriptome Analysis Revealed the Symbiosis Niche of 3D Scaffolds to Accelerate Bone Defect Healing
Source: Adv Sci (Weinh). 2022 Jan 18;9(8):2105194. doi: 10.1002/advs.202105194 (PMC8922091; doi:10.1002/advs.202105194)
Supplement: Supplementary file 1 — Supporting Information [file ADVS-9-2105194-s009.pdf]

ADVANCED SCIENCE
Open Access

Supporting Information

for *Adv. Sci.*, DOI 10.1002/advs.202105194

Transcriptome Analysis Revealed the Symbiosis Niche of 3D Scaffolds to Accelerate Bone Defect Healing

Ce Ji, Minglong Qiu, Huitong Ruan, Cuidi Li, Liang Cheng, Juan Wang, Changwei Li, Jin Qi, Wenguo Cui* and Lianfu Deng**

ADVANCED SCIENCE
Open Access

Supporting Information

for *Adv. Sci.*, DOI: 10.1002/advs.202105194

Transcriptome analysis revealed the symbiosis niche of 3D scaffolds to accelerate bone defect healing

Ce Ji, Minglong Qiu, Huitong Ruan, Cuidi Li, Liang Cheng, Juan Wang, Changwei Li, Jin Qi, Wenguo Cui**, and *Lianfu Deng**

Supporting Information

Title

Transcriptome analysis revealed the symbiosis niche of 3D scaffolds to accelerate bone defect healing

Ce Ji, Minglong Qiu, Huitong Ruan, Cuidi Li, Liang Cheng, Juan Wang, Changwei Li, Jin Qi, Wenguo Cui*, Lianfu Deng**

Dr. C. Ji, M. Qiu, Dr. H. Ruan, Dr. C. Li, Dr. L. Cheng, Dr. J. Wang, Dr. C. Li, Prof. J. Qi, Prof. W. Cui, Prof. L. Deng

Department of Orthopaedics, Shanghai Key Laboratory for Prevention and Treatment of Bone and Joint Diseases, Shanghai Institute of Traumatology and Orthopaedics, Ruijin Hospital, Shanghai Jiao Tong University School of Medicine, 197 Ruijin 2nd Road, Shanghai 200025, P. R. China.

Email addresses: jinjin838@hotmail.com (J. Qi), wgcui80@hotmail.com (W. Cui), lf_deng@126.com (L. Deng)

Figure S1.

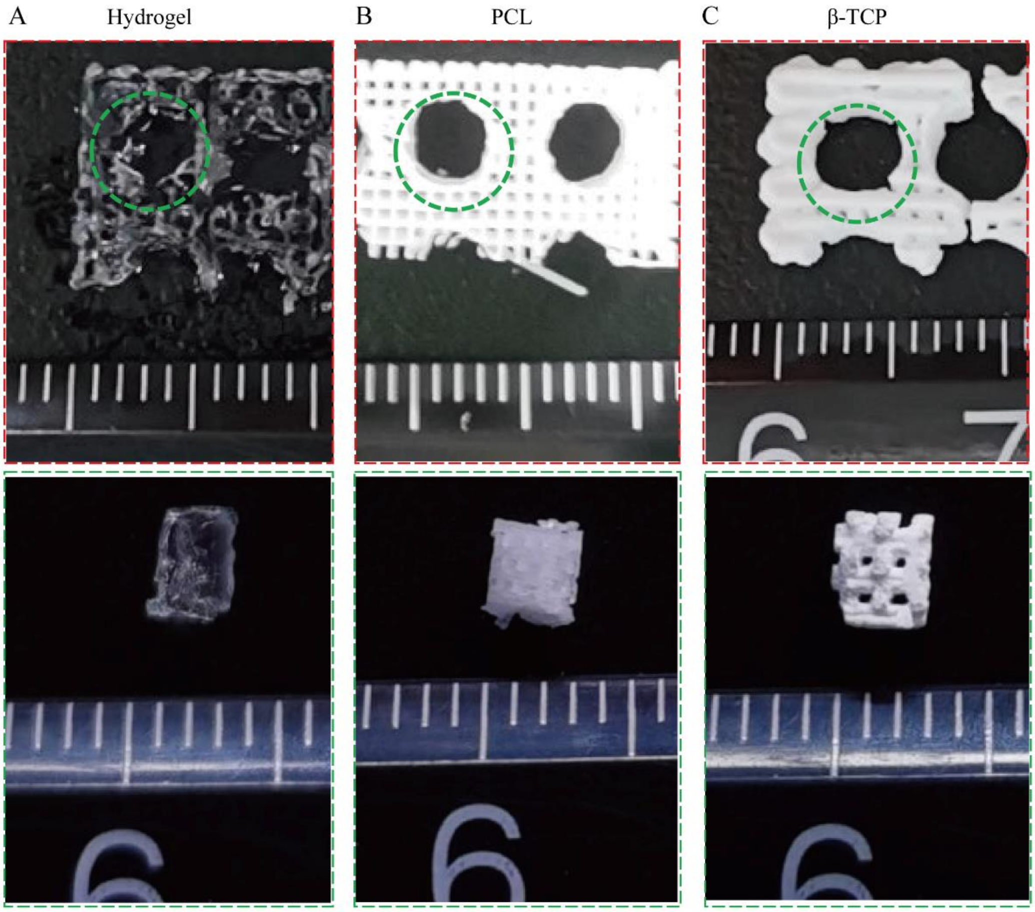

Figure S1. Characterization of the 3D bioprinting scaffolds. The implantation cylinder (diameter: 3mm, height: 3mm) was cropped from cube scaffolds (20 *20 *3 mm). (A) GelMA hydrogel scaffold. (B) PCL scaffold. (C) β-TCP scaffolds.

Figure S2.

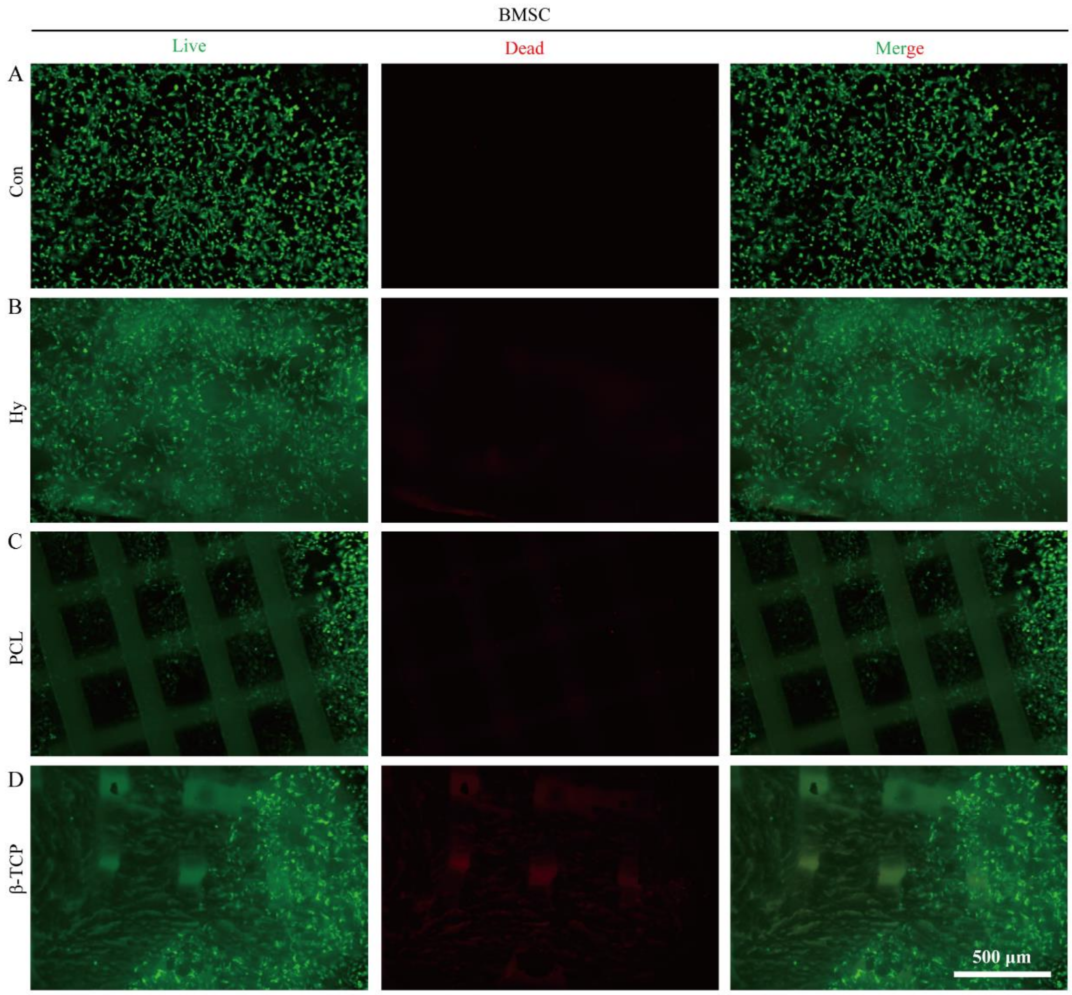

Figure S2. Biocompatibility of the hydrogel, PCL and β-TCP scaffolds (BMSC cells). Live and dead detection of BMSC cocultured with no scaffold (A), hydrogel (B), PCL (C) and β-TCP scaffolds (D) at 3 days.

Figure S3.

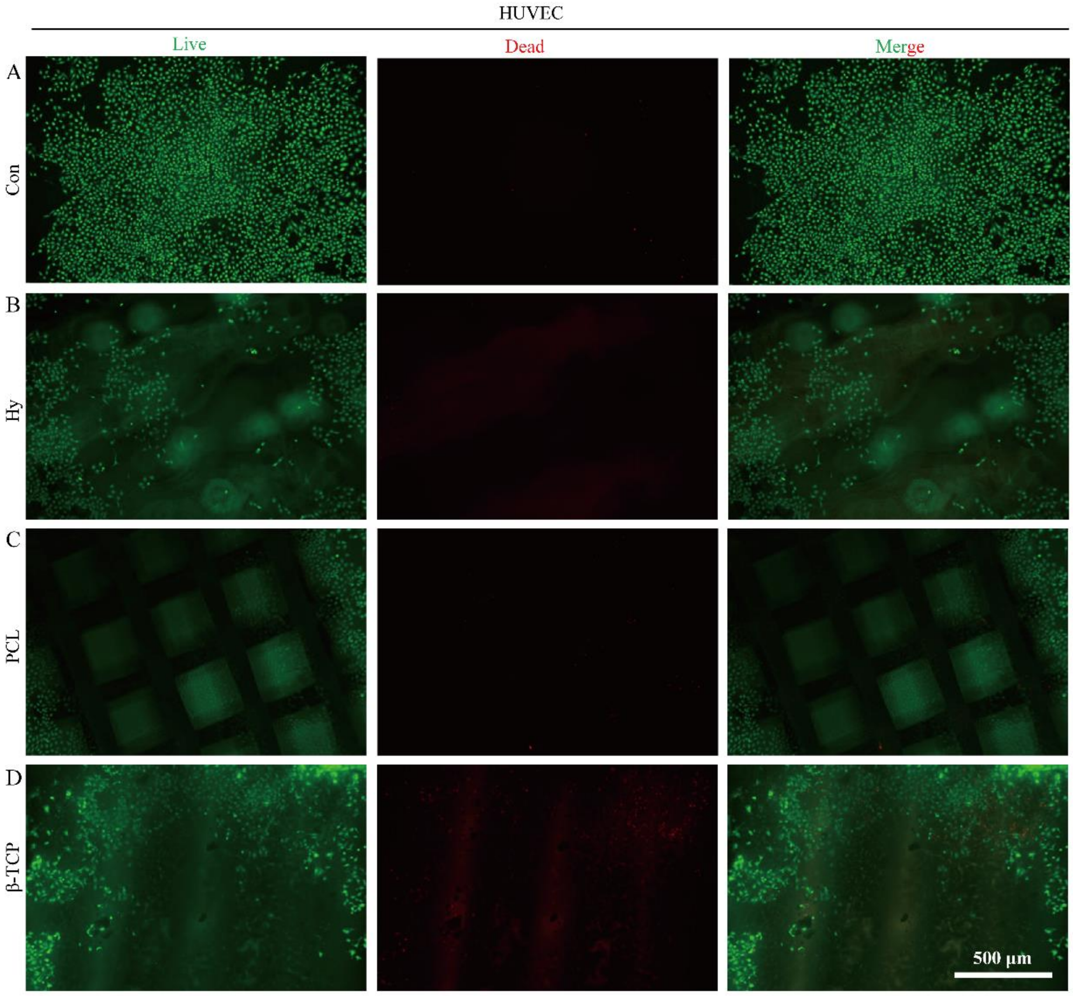

Figure S3. Biocompatibility of the hydrogel, PCL and β-TCP scaffolds (HUVEC cells). Live and dead detection of HUVEC cocultured with no scaffold (A), hydrogel (B), PCL (C) and β-TCP scaffolds (D) at 3 days.

Figure S4.

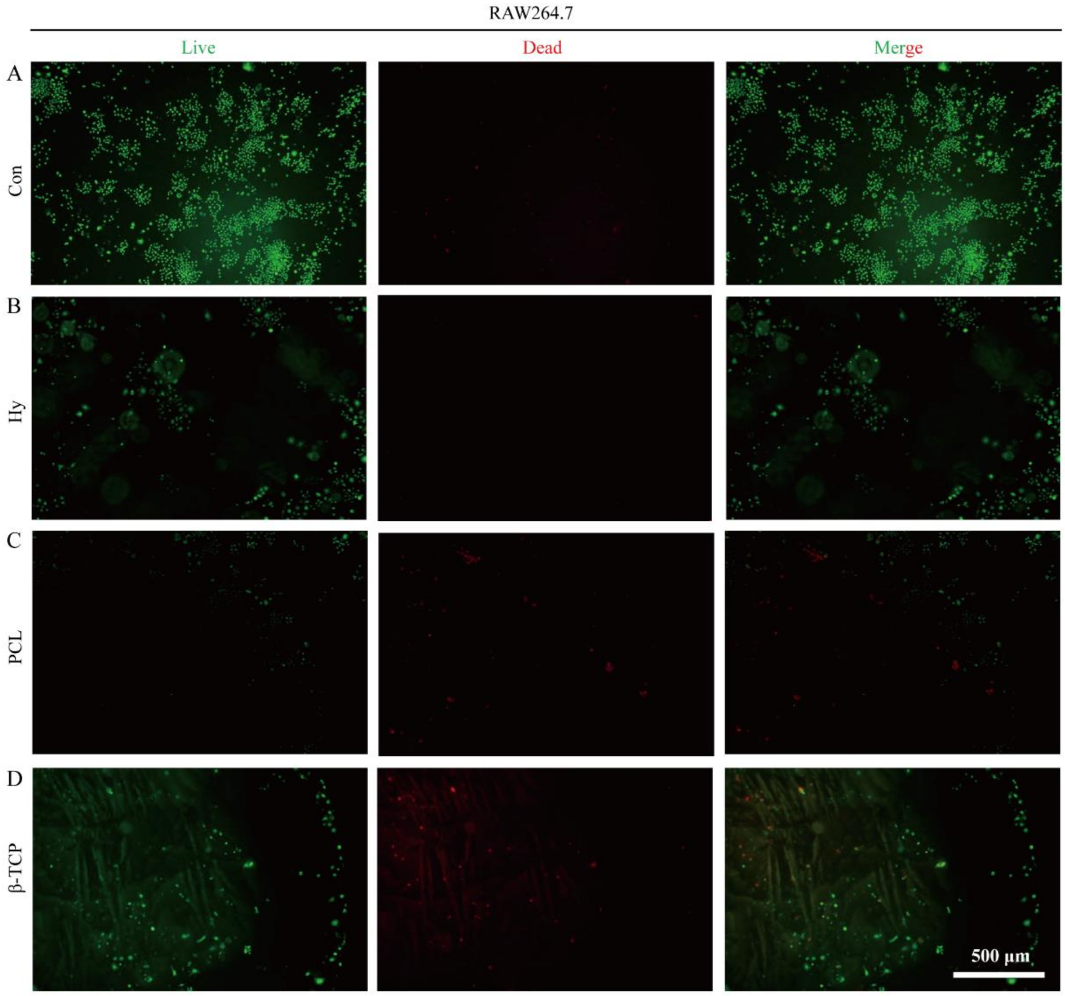

Figure S4. Biocompatibility of the hydrogel, PCL and β-TCP scaffolds (RAW264.7 cells). Live and dead detection of RAW264.7 cocultured with no scaffold (A), hydrogel (B), PCL (C) and β-TCP scaffolds (D) at 3 days.

Figure S5.

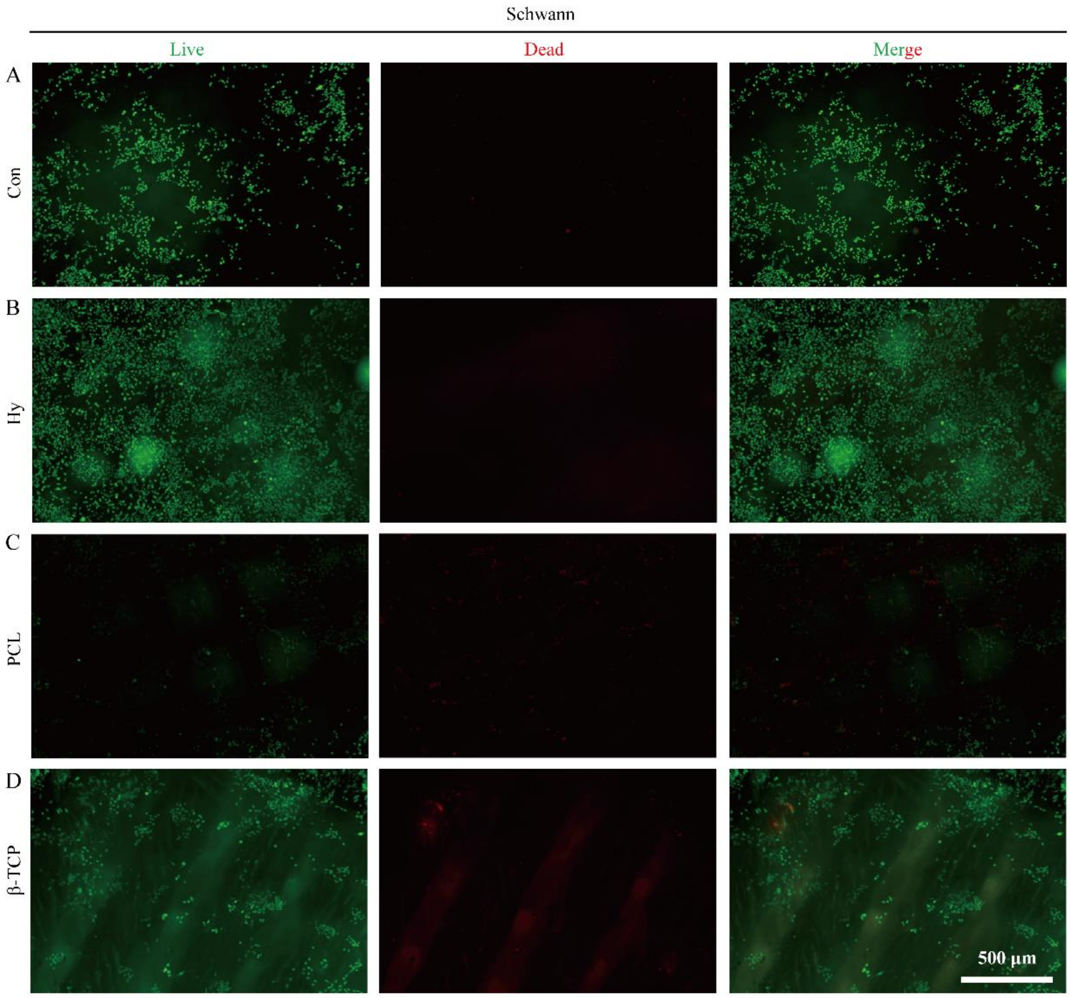

Figure S5. Biocompatibility of the hydrogel, PCL and β-TCP scaffolds (Schwann cells). Live and dead detection of Schwann cocultured with no scaffold (A), hydrogel (B), PCL (C) and β-TCP scaffolds (D) at 3 days.

Figure S6.

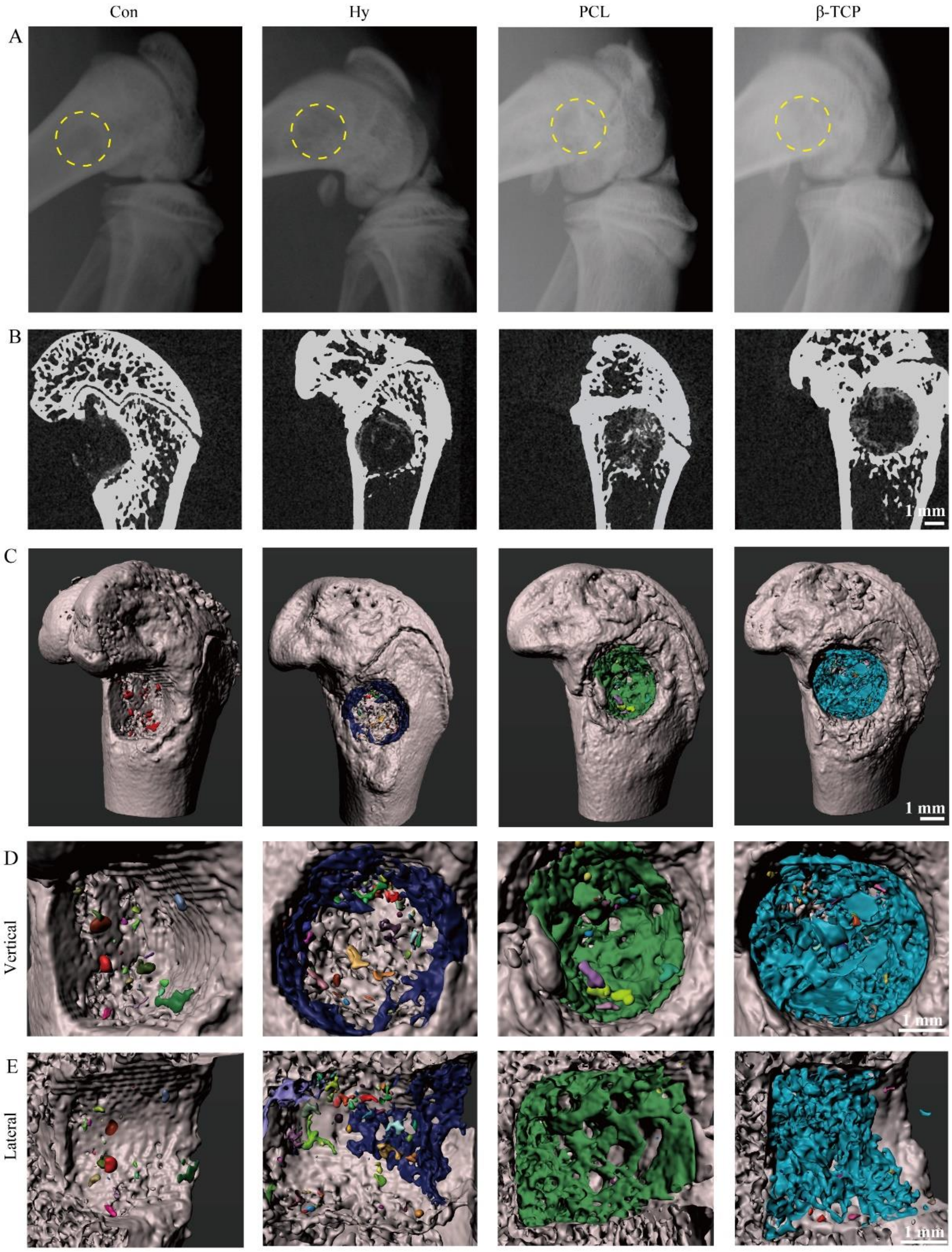

Figure S6. Image and reconstruction analysis of osteogenesis *in vivo*. The hydrogel, PCL and β-TCP scaffolds were implanted into critical size defect model and the new bone were detected and reconstructed by X ray (A), MicroCT (B) and Imaris (C). (D) Vertical view of 3D bone construction in control, hydrogel, PCL and β-TCP scaffolds implantation groups at 2 weeks. (E) Lateral view of 3D bone construction at 2 weeks.

Figure S7.

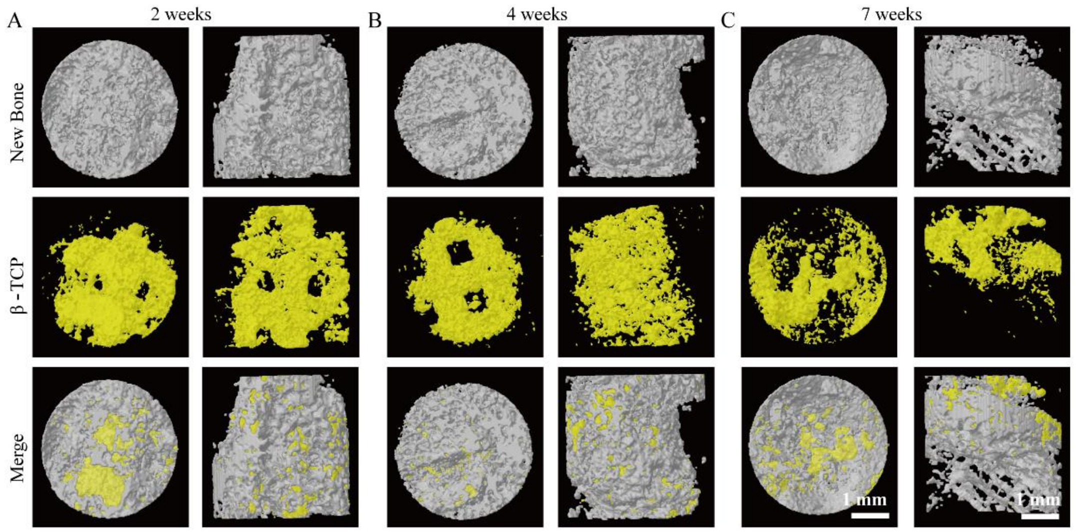

Figure S7. Reconstruction of β-TCP scaffold and new bone. The β-TCP scaffold implanted group were scanned by microCT and the results were reconstructed by microCT software. The β-TCP scaffold and new bone were labelled by yellow and white by density characteristic at 2 weeks (A), 4 weeks (B) and 7 weeks (C).

Figure S8.

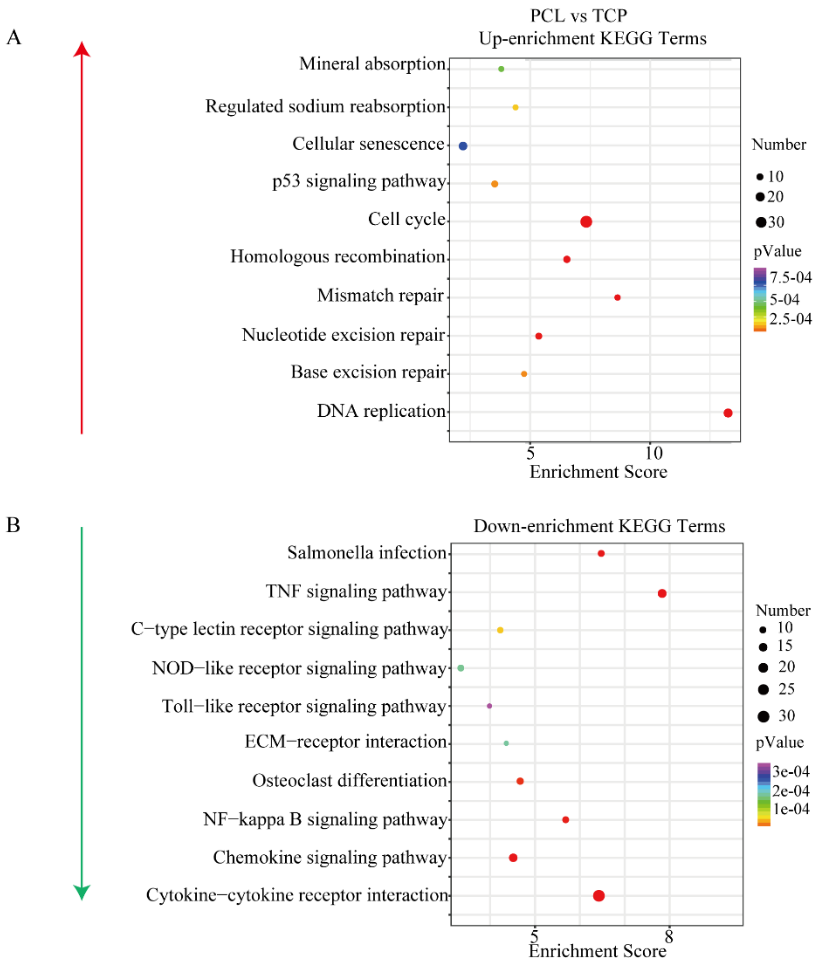

Figure S8. KEGG enrichment analysis of DEGs in β-TCP group compared with PCL group. (A) The top 10 up enrichment KEGG pathways. (B) The top 10 down enrichment KEGG pathways.

Table S1.

Table S1. Primer sequence used for RT-qPCR analysis.

Genes	Primer sequence (5′–3′)	NCBI ID
Ifi27l2b F	TTGGACTCTCCACAATAACCAAC	NM_206846.2
Ifi27l2b R	CTGCTGCAATTCCACCTCC	
Cd5l F	GGGGTTGACTGCAACGGAA	NM_001025685.1
Cd5l R	GGCCATCTACTAGACGCACA	
Lilrb3a F	CCATGCCACTAGGTGGACTG	NM_001313924.1
Lilrb3a R	GAATTTTCCCCGATGTCTTCGT	
Cxcl13 F	GGCCACGGTATTCTGGAAGC	NM_001017496.1
Cxcl13 R	GGGCGTAACTTGAATCCGATCTA	
Ccl24 F	ATTCTGTGACCATCCCCTCAT	NM_001013045.1
Ccl24 R	TGTATGTGCCTCTGAACCCAC	
Col2a1 F	GGGAATGTCCTCTGCGATGAC	NM_012929.1
Col2a1 R	GAAGGGGATCTCGGGGTTG	
Mgp F	GGCAACCCTGTGCTACGAAT	NM_012862.2
Mgp R	CCTGGACTCTCTTTTGGGCTTTA	
Col1a2 F	GTAACTTCGTGCCTAGCAACA	NM_053356.2
Col1a2 R	CCTTTGTCAGAATACTGAGCAGC	
Col11a1 F	ACAAAACCCCTCGATAGAAGTGA	NM_013117.1
Col11a1 R	CTCAGGTGCATACTCATCAATGT	
Acan F	CCTGCTACTTCATCGACCCC	XM_032893390.1
Acan R	AGATGCTGTTGACTCGAACCT	
Gapdh F	AAGTTCAACGGCACAGTCAAG	NM_017008.4
Gapdh R	ACATACTCAGCACCAGCATCA	
